# Preoperative Vitamin D as Predictor of MRONJ: A Retrospective Multivariate Analysis

**DOI:** 10.3390/jcm15051712

**Published:** 2026-02-24

**Authors:** Raluca Maracineanu, Serban Talpos-Niculescu, Marilena Dinuți, Marius Pricop, Roxana Folescu, Alexandra-Denisa Semenescu, Ivona Mihaela Hum

**Affiliations:** 1 Doctoral School, “Victor Babeș” University of Medicine and Pharmacy, 300062 Timisoara, Romania; raluca.zibileanu@umft.ro (R.M.); ivona.ursu@umft.ro (I.M.H.); 2 Discipline of Oral and Maxillofacial Surgery, Faculty of Dental Medicine, “Victor Babeș” University of Medicine and Pharmacy, 300062 Timisoara, Romania; pricop.marius@umft.ro; 3 Department of Biochemistry and Pharmacology, Discipline of Biochemistry, “Victor Babeș” University of Medicine and Pharmacy, 300041 Timisoara, Romania; motoc.marilena@umft.ro; 4 Department of Balneology, Medical Recovery and Rheumatology, Family Medicine University Clinic, Center for Preventive Medicine, Center for Advanced Research in Cardiovascular Pathology and Hemostaseology, “Victor Babeș” University of Medicine and Pharmacy, 300041 Timisoara, Romania; folescu.roxana@umft.ro; 5 Department of Toxicology, Drug Industry, Management, Marketing and Dermatopharmacy, Faculty of Pharmacy, “Victor Babeș” University of Medicine and Pharmacy, 300041 Timisoara, Romania; alexandra.scurtu@umft.ro; 6 Research Centre for Pharmaco-Toxicological Evaluation, “Victor Babeș” University of Medicine and Pharmacy, 300041 Timisoara, Romania

**Keywords:** MRONJ, vitamin D, β-CTX, dental extractions, antiresorptive therapy

## Abstract

**Background**: Medication-related osteonecrosis of the jaw (MRONJ) is a grave complication in patients with cancer treated with antiresorptive agents, particularly after invasive dental procedures. Identifying reliable clinical factors to assess MRONJ risk remains a clinical challenge. **Methods**: The retrospective observational study comprised 61 oncologic patients undergoing dental extractions during antiresorptive therapy. Preoperative serum levels of 25-hydroxyvitamin D and β-C-terminal telopeptide cross-link (β-CTx), along with relevant clinical variables, were measured. The analyses included comparative tests, multivariate logistic regression to detect independent predictors of MRONJ, and ROC curve analysis to assess the model’s predictive performance. **Results**: MRONJ was diagnosed in 18 patients (29.5%). Low preoperative vitamin D levels were significantly associated with MRONJ and remained an independent predictor in the multivariate analysis (OR = 8.74, *p* = 0.005). The mandibular extraction site was also identified as a significant risk factor (OR = 7.94, *p* = 0.007). In contrast, β-CTX levels, age, sex, cancer type, and the number of extracted teeth did not show a significant link to MRONJ development in this cohort. The comprehensive multivariate model demonstrated good discrimination capacity (AUC = 0.806). **Conclusions**: Preoperative vitamin D deficiency is an important independent predictor of MRONJ after dental extractions in patients with cancer receiving antiresorptive agents. Integrating metabolic biomarkers and clinical variables into predictive models may improve risk assessment and support the development of more effective preoperative prevention strategies.

## 1. Introduction

Medication-related osteonecrosis of the jaw (MRONJ) is a severe complication caused by the use of antiresorptive or antiangiogenic drugs, leading to gradual bone damage in the oro-maxillofacial region [1,2,3,4].

Specifically, according to the American Association of Oral and Maxillofacial Surgeons (AAOMS), MRONJ is defined as an oral dehiscence with exposed necrotic bone lasting more than 8 weeks in patients who have not been diagnosed with metastases, have not received radiotherapy, but are currently or previously treated with antiresorptive medications like bisphosphonates or denosumab, or antiangiogenic therapy [2,5]. These drug classes offer significant benefits for the primary condition; therefore, preventing and treating MRONJ is crucial [6].

The main mechanisms contributing to MRONJ progression include: (i) inhibition of bone remodeling, which decreases osteoclast activity through apoptosis; (ii) infection and inflammation at the tooth extraction site, which cause bone resorption; and (iii) inhibition of vascular endothelial growth factor, which suppresses angiogenesis [7,8,9,10,11,12].

Since MRONJ was first identified in 2003, there has been considerable progress in its management. Despite these advancements, this topic remains of great interest to clinicians because MRONJ does not result from a single cause, indicating a multifactorial origin [9,13,14]. This oral disease significantly impacts quality of life; therefore, a clear understanding of the pathogenesis and risk factors involved in MRONJ is essential for implementing preventive measures [15,16].

According to the literature, the strongest hypothesis regarding the etiology and pathophysiology of MRONJ is the inhibition of bone remodeling associated with chronic inflammation, leading to osteonecrosis [17,18,19].

Various studies have examined the role of biomarkers in MRONJ progression [20,21,22]; however, further detailed research is needed.

Serum β-C-terminal telopeptide cross-link (β-CTx) is a blood biomarker for bone remodeling. For the past 10 years, it has been considered a prognostic factor and possible predictor of MRONJ in patients undergoing dental procedures while on antiresorptives. In 2007, Mark et al. reported that patients treated with bisphosphonates and dental interventions may be at risk for MRONJ, with risk classified by β-CTx levels: high (<100 pg/mL), moderate (100–150 pg/mL), and low (>150 pg/mL). As a result, clinicians have measured serum β-CTx levels in these patients before dental procedures [23,24,25,26]. Nevertheless, the reliability of β-CTx as an MRONJ predictor remains debated, as current evidence does not clearly support or refute its predictive value [27,28,29].

Vitamin D is critical for preventing osteoporosis and certain cancers, such as breast cancer. It also plays a substantial role in managing patients with MRONJ, and may serve as a risk factor for the development of MRONJ in patients with cancer treated with antiresorptive medication. Research indicates that vulnerable patients, including those with prostate cancer and multiple myeloma, often have a vitamin D deficiency, which increases their risk for MRONJ. Consequently, maintaining adequate vitamin D levels is essential for at-risk populations [30,31,32,33,34].

In the context of ongoing controversies about the usefulness of metabolic biomarkers in assessing the risk of MRONJ, the present study aimed to determine the association between preoperative levels of 25-hydroxyvitamin D and β-CTx and the development of MRONJ in patients with cancer undergoing antiresorptive therapy and tooth extractions. In parallel, the influence of clinically relevant factors, including extraction location, age, sex, number of extracted teeth, and type of malignancy, was analyzed to investigate their impact on the bone healing process. In addition, a multivariable logistic regression model was developed to identify independent predictors of MRONJ and to evaluate its predictive capacity using the ROC curve, to improve preoperative risk assessment strategies for MRONJ.

## 2. Materials and Methods

### 2.1. Study Design

We conducted a retrospective observational study of 61 oncologic patients receiving antiresorptive therapy who underwent dental extractions. Clinical and laboratory records served as the primary sources of data.

The research was conducted in accordance with the principles outlined in the Declaration of Helsinki. The study took place at the Department of Oral and Maxillofacial Surgery, Municipal Emergency Hospital, Victor Babeș University of Medicine and Pharmacy, Timișoara, over 24 months. The Independent Ethics Committee of the University of Medicine and Pharmacy “Victor Babeș”, Timișoara, Romania, granted ethical approval (no. 51/2 October 2023).

Inclusion criteria:

- diagnosis of malignancy;
- exposure to zoledronic acid;
- patients in whom bisphosphonate was discontinued at least 2 months before dental extraction;
- at least one dental extraction;
- preoperative serum 25-hydroxyvitamin D (25-OH-vitamin D) and β-C-terminal telopeptide (β-CTx) measured before extraction;
- documented follow-up at 8 weeks.

Exclusion criteria:

- missing primary biomarkers or outcome;
- non-oncologic antiresorptive indications;
- concomitant jaw pathology preventing outcome assessment;
- pregnancy or breastfeeding;
- patients who were given oral bisphosphonates;
- patients who had undergone radiotherapy to the head and neck;
- patients with oral or dental surgical treatments in the last year;
- patients with impacted wisdom teeth or ankylosed dental remains.

### 2.2. Participants, Clinical Variables, and Procedures

The sample comprised 61 patients with complete preoperative biochemical data and outcomes recorded at 8 weeks postoperatively. The study focused on whether MRONJ was present or absent at 8 weeks post-dental extraction.

Before surgery, each patient’s medical history was thoroughly reviewed, and imaging assessments, including Cone Beam Computed Tomography or panoramic radiography, were performed.

Serum levels of 25-OH-vitamin D and β-CTX were determined from preoperative peripheral blood samples collected under standardized conditions. The analyses were performed using the electrochemiluminescence immunoassay method, according to the manufacturer’s instructions. The samples were analyzed in an accredited laboratory, and the results were expressed in µg/L for 25-OH-vitamin D and pg/mL for β-CTX. The results, along with other variables, were extracted from the electronic medical records.

Metabolic biomarker values were categorized using clinically relevant cutoffs: 25-OH-vitamin D: <30 µg/L (deficient) vs. >30 µg/L (normal); and β-CTx: <150 pg/mL (high or moderate risk of MRONJ) vs. >150 pg/mL (low risk). Age (years), sex (female/male), extraction site (mandible vs. maxilla), number of extracted teeth, and malignancy type were also recorded and assessed as potential predictors of MRONJ.

Twenty-four hours before the surgical procedure, patients received a prophylactic antibiotic consisting of amoxicillin and clavulanic acid (1 g every 12 h) with metronidazole (250 mg every 12 h). In addition, morning and evening oral rinses with a 0.2% chlorhexidine antiseptic solution were required. Patients allergic to beta-lactams were prescribed clindamycin (600 mg every 8 h).

Dental extractions were conducted using techniques designed to minimize trauma. A single surgeon used standard oral surgery methods to reduce variations that could affect the outcome. All extractions were performed under local anesthesia with articaine hydrochloride containing epinephrine at a concentration of 1:100,000 or 1:200,000 Ubistesin Forte (3M ESPE, Seefeld, Germany), depending on the patient’s underlying condition and the required hemostatic effect. Under local anesthesia, the syndesmosis of the tooth was performed; the tooth was dislodged with elevators and was subsequently extracted from the arch using forceps. The post-extraction alveoli were irrigated with saline, local hemostasis was performed, and a local hemostatic material ROEKO Gelatamp (Coltène/Whaledent GmbH & Co. KG, Langenau, Germany) was applied intraalveolarly. The post-extraction socket was closed with a tension-free muco-periosteal local flap sutured with a non-absorbable polyamide monofilament, 4/0 (Supramid, B Braun, Rubi, Spain). The closure technique was performed according to the surgical principles published by Ruggiero et al. in 2022 [2], which aim to achieve complete mucosal closure, without tension, to avoid bone exposure in the oral cavity. The antibiotic regimen administered the day before the dental extractions was also applied 6 days after the intervention. The sutures were removed 14 days after surgery, and follow-up of the post-extraction wound was conducted at 8 weeks.

The extracted teeth were irretrievable, periodontal, or with periapical lesions that could not be treated cautiously. All extractions were performed as conservatively as possible, without damaging the adjacent alveolar bone. Patients with impacted wisdom molars or ankylosed dental remains that required milling of the adjacent bone were not included in the study. These types of subjects may compromise the accuracy of the final results due to the application of thermal trauma with the milling cutters to the bone and the potential for osteonecrosis induced by them. No alveoloplasty or local hard tissue debridement was performed in any case, except for soft tissue debridement in cases where periodontal or periapical lesions were present.

All patients agreed to the procedure, provided informed consent, and received detailed post-extraction instructions, including dietary and oral hygiene, as well as information about possible complications. Therefore, patients were instructed to avoid brushing directly over the intervention site for the first 24 h, then gradually resume oral hygiene with gentle brushing. Rinsing the oral cavity with an antiseptic solution containing 0.2% chlorhexidine was recommended after 72 h according to the clinical protocol. From a dietary point of view, patients were advised to follow a soft diet at moderate temperature, avoiding hard or very hot foods during the first postoperative days, to prevent alveolar traumatization. They were also advised to avoid smoking and alcohol consumption in the immediate postoperative period [35].

The diagnosis of MRONJ was established according to the AAOMS criteria. Cases were considered MRONJ if at the 8-week follow-up there was gingival dehiscence with bone exposure, signs of superinfection, or the presence of a dental fistula [2]. Thus, groups were divided into “MRONJ at 8 weeks” or “normal healing” if those indicators were absent.

The statistical analysis was performed by the clinical pharmacist of the group, together with the attending physician. The attending physician, a specialist in oral and maxillofacial surgery, performed the initial clinical assessment, extractions, and re-evaluation at 8 weeks. The physician involved in establishing the diagnosis had access to the patient’s clinical information, but serum biomarker levels were analyzed separately, and statistical interpretation was performed subsequently.

### 2.3. Statistical Analysis

Statistical analyses were conducted using JASP software (version 0.18.3; JASP Team, University of Amsterdam, Amsterdam, The Netherlands; https://jasp-stats.org). Continuous variables were recorded as mean ± standard deviation (SD) or median with interquartile range (Q1–Q3). Categorical variables were exhibited as absolute numbers and percentages. Comparisons between the MRONJ at 8 weeks and normal-healing groups were performed using the Mann–Whitney U test. Associations between categorical predictors and MRONJ incidence were assessed using Fisher’s exact test. Odds ratios (ORs) with 95% confidence intervals (CIs) were calculated to assess effect size. A multivariate logistic regression model was utilized to detect independent predictors of MRONJ. Discriminative ability was assessed using the receiver operating characteristic (ROC) curve and the area under the curve (AUC). Statistical significance was set at *p* < 0.05.

## 3. Results

Table 1 shows the baseline characteristics of the study group. There were 61 patients with cancer, with an average age of 64.84 years (±9.41), ranging from 42 to 82 years old. Most were women (37 cases, 60.7%), and 24 were men (39.3%).

Breast cancer was the most common, found in 25 patients (40.98%). Prostate cancer was next, with 19 patients (31.15%). Kidney and brain cancers were each seen in 5 patients (8.2%). Three patients (4.92%) had gastric cancer, and broncho-pulmonary cancer and multiple myeloma were each found in 2 patients (3.28%).

Regarding the location of dental interventions, 35 dental extractions (57.38%) were performed in the mandible, while 26 (42.62%) were performed in the maxilla. Also, the average number of teeth extracted per patient was 2.69 ± 1.65.

Preoperative serum biomarker analysis revealed a median β-CTx value of 332 pg/mL, with an interquartile range of 183 to 511 pg/mL. Regarding vitamin D status, the median value was 27.07 ng/mL, with an interquartile range of 20.62 to 35.17 ng/mL.

At the 8-week post-procedure evaluation, 43 patients (70.49%) healed normally, whereas 18 patients (29.51%) developed MRONJ.

As we observed, 60.7% of the 61 patients were women, and 39.3% were men. Among these patients, 24.32% of women (9 out of 28) developed MRONJ, compared to 37.5% of men (9 out of 15). No statistically significant link between gender and MRONJ occurrence was found (Fisher’s exact test, *p* = 0.389), as the same number of female and male patients (9 each) developed MRONJ. The analysis showed a trend toward better outcomes in women, with a higher likelihood of normal healing, but this difference was not statistically significant (OR = 1.87; 95% CI: 0.61–5.7).

The influence of age on MRONJ development is shown in Table 2. The graphical analysis did not reveal significant differences in age distribution between oncological patients with MRONJ and those with normal healing (Mann–Whitney U test, *p* = 0.645). The medians are similar (64 vs. 68), and the interquartile ranges overlap considerably (55–74 vs. 59–73). Although the MRONJ group appears to include older patients, the mean is actually lower.

Breast cancer (41%), followed by prostate cancer (31.1%), was the most common primary diagnosis in our study, which was directly related to the higher number of female patients receiving antiresorptive therapy. The distribution of cancer types is similar between the MRONJ and non-MRONJ groups. Among the 18 patients with MRONJ, 33.33% had prostate cancer, 27.78% had breast cancer, and 16.67% had kidney cancer. The remaining types of cancer each accounted for about 5.6% (brain, bronchopulmonary, stomach cancer, and multiple myeloma). There was no significant association between any cancer type and MRONJ (Chi-square test, *p* = 0.622).

Of 35 patients who underwent mandibular extractions, 15 (42.86%) developed MRONJ. In comparison, only 3 (11.54%) out of 26 oncological patients who had maxillary extractions developed the condition. Mandibular extractions were associated with a much higher risk of MRONJ, consistent with known anatomical susceptibility (Fisher’s exact test, *p* = 0.011). The risk of developing MRONJ was approximately 5.6 times greater after mandibular than after maxillary extractions (OR = 5.59, 95% CI: 1.31–34.47), suggesting that location may be a key risk factor.

Regarding the influence of the number of extracted teeth, our analysis (Table 3) shows no statistically significant difference between the MRONJ and non-MRONJ groups (Mann–Whitney U test, *p* = 0.929). The values were almost identical; the median was the same in both cases (2 teeth), and the mean for patients with MRONJ was 2.68 ± 1.53, compared to 2.7 ± 1.71 for those with normal healing.

Furthermore, preoperative serum levels of β-CTX and 25-OH-vitamin D were assessed as continuous variables to identify potential biological differences between the MRONJ and normal-healing groups. The results of this analysis are exposed in Table 4.

There was no statistically significant difference in preoperative β-CTx values between patients who developed MRONJ and those with normal healing (Mann–Whitney U test, *p* = 0.621). The mean and median values are slightly higher in the MRONJ group, but the difference is minor, not clinically significant. Furthermore, a large heterogeneity of β-CTx values is observed, indicated by a high SD. In the case of vitamin D, patients who developed MRONJ had significantly lower preoperative 25-OH-vitamin D levels than the normal-healing group (Mann–Whitney U test, *p* = 0.001). The difference between the two groups is statistically significant. To determine the clinical relevance of the thresholds used in current practice, preoperative β-CTX and 25-OH-vitamin D levels were classified into risk categories, analyzed as categorical variables, and their association with MRONJ incidence at 8 weeks postoperatively was expressed as odds ratios. The results are stated in Table 5.

Antiresorptive-treated patients with cancer with preoperative vitamin D deficiency (<30 µg/L) had a significantly higher risk of developing MRONJ 8 weeks after tooth extraction (Fisher’s exact test, *p* = 0.006, OR = 6.32, 95% CI: 1.59–25.05). In contrast, no significant association was observed between preoperative β-CTx levels and MRONJ incidence (Fisher’s exact test, *p* = 0.737, OR = 0.66, 95% CI: 0.16–2.75, *p* = 0.737).

A multivariate logistic regression model was further performed to assess the predictive value of metabolic biomarkers for the development of MRONJ. The model included preoperative 25-OH-vitamin D and β-CTx levels (Table 6).

Patients with preoperative 25-OH-vitamin D levels under 30 µg/L had a much higher risk of MRONJ (OR = 6.33, 95% CI: 1.59–25.19, *p* = 0.009). In contrast, there was no significant link between β-CTx levels and MRONJ (OR = 0.65, 95% CI: 0.14–2.97, *p* = 0.579). The predictive model had moderate accuracy (AUC = 0.709). Our findings indicate that β-CTx had no predictive value in this data set and under the measurement conditions employed.

To create a clinically applicable prediction model, we additionally introduced relevant variables, tooth extraction location, and age, to the initial metabolic biomarker-based model (Table 7). This complex multivariate model assessed the contribution of both biological and clinical factors to MRONJ.

The extensive multivariable logistic regression analysis showed that preoperative 25-OH-vitamin D deficiency (<30 µg/L) was independently associated with a higher risk of MRONJ (OR = 8.74, 95% CI: 1.96–39.03, *p* = 0.005). Also, extraction at the mandibular level was an independent risk factor (OR = 7.94, 95% CI: 1.75–35.99, *p* = 0.007). On the other hand, β-CTx levels were not significantly linked with the development of MRONJ (OR = 0.82, 95% CI: 0.15–4.47, *p* = 0.815), and the age of the patients did not have a significant impact on the risk of MRONJ (OR = 0.99, 95% CI: 0.93–1.06, *p* = 0.866) in this data set. The complex multivariate logistic regression model recorded an AUC of 0.806, demonstrating good discriminative capacity.

The extended model outperformed the model that used only 25-OH-vitamin D and β-CTX, increasing the AUC from 0.709 to 0.806. This improvement indicates a greater ability to distinguish between the MRONJ and normal-healing groups.

The extended multivariable logistic model indicated that preoperative vitamin D deficiency and mandibular extraction site were independent predictors of MRONJ. At the same time, β-CTX level and patient age were not significantly linked with the postoperative development of MRONJ in our cohort. The model (AUC = 0.806) supports the clinical relevance of both metabolic and procedural factors in assessing MRONJ risk.

**Figure 1:**
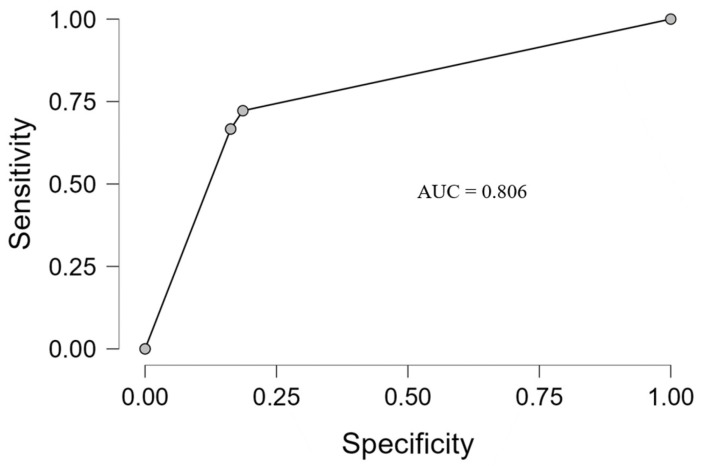
ROC curve of the multivariate logistic regression model for the prediction of MRONJ occurrence.

To evaluate the discrimination ability of the comprehensive multivariate logistic regression model, ROC curve analysis was conducted (Figure 1).

The ROC analysis illustrated a good predictive ability for the multivariate model (AUC of 0.806). The model had a sensitivity of 66.7% (indicating ≈ 67% of patients will develop MRONJ) and a specificity of 83.7% (classifying ≈ 84% of patients with normal healing), demonstrating good capability to differentiate the two groups. Sensitivity and specificity were calculated using the default probability cut-off of 0.5.

## 4. Discussion

MRONJ has attracted attention in recent years because of the increasing number of cases [36]. Therefore, effective management of MRONJ needs to be defined. There are numerous risk factors, including medication-related, local, and systemic factors, that may contribute to the development of this oral pathology [3].

Treatment measures are contentious because clinical evidence regarding the pathogenesis of MRONJ is limited. Current treatment aims to relieve pain, avoid infection, and handle bone resorption and necrosis. Prevention is important in reducing the risk of MRONJ [37,38]. A thorough medical examination of patients can significantly reduce the risk of developing MRONJ [39]. Risk assessment of each patient is necessary to control the development of MRONJ [40].

In the current study, the main results indicated that preoperative 25-hydroxyvitamin D deficiency is a significant independent predictor of MRONJ after dental extractions in patients with cancer treated with antiresorptive drugs. Moreover, mandibular extractions were associated with an increased risk of MRONJ. On the other hand, β-CTX levels did not significantly influence MRONJ in the analyzed cohort. The findings underscore the significance of evaluating metabolic biomarkers and anatomical factors during preoperative risk stratification. The results offer a clinically relevant perspective on the role of vitamin D in bone metabolism.

International guidelines recommend measuring serum 25-OH-vitamin D levels to determine vitamin D status, which is essential for both bone health and immune system function. Serum vitamin D levels can be linked to clinical events, including fracture risk and bone mineralization [41].

Serum 25-OH-vitamin D levels < 10 ng/mL are classified as severe vitamin D deficiency; levels < 30 ng/mL are considered insufficient (hypovitaminosis D), while values > 30 ng/mL represent a normal level of vitamin D in the body [42].

Vitamin D deficiency represents a global health concern. Vitamin D has implications in inflammatory, neurodegenerative diseases, and cancer [43,44,45,46]. Some studies express the fact that 35–40% of patients with breast carcinoma and multiple myeloma, respectively, had a vitamin D deficiency [47,48]. Furthermore, 25-OH-vitamin D plays a key role as a modulator of immune responses [49].

The vitamin D status in patients with MRONJ has not been clearly established. In many situations, patients who present with numerous clinical or local risk factors for MRONJ have low vitamin D levels [42].

In our study, preoperative 25-OH-vitamin D levels were evaluated and showed significant differences between patients with MRONJ and those with normal healing. Patients who developed MRONJ had significantly lower levels than patients with a favorable outcome after dental interventions. The categorical analysis showed that patients with preoperative vitamin D deficiency had an increased rate of MRONJ. The logistic regression model also found that having a vitamin D level below 30 µg/L was a significant independent predictor of MRONJ. These results suggest that vitamin D has an important role in bone healing after dental extractions.

Our results, which show that vitamin D deficiency is involved in the development of MRONJ, are supported by studies from other research groups. In a retrospective study, Heim et al. demonstrated that patients with stage 2 osteonecrosis had much lower vitamin D levels than those without exposed bone. Furthermore, vitamin D levels were lower among patients on antiresorptive treatment (20.49 ng/mL) than among those without this medication (29.5 ng/mL). These data suggested the need for adequate vitamin D supplementation in patients treated with antiresorptives [42].

The group led by Michalak et al. found that vitamin D supplementation before or during teeth extraction decreased the risk of severe MRONJ (OR = 68.57; *p* < 0.001). Patients with vitamin D deficiency received a 10,000 IU dose for 1 month, after which the dose was decreased to 5000 IU/day for 3 to 6 months to maintain vitamin D levels < 100 ng/mL and reduce toxicity. However, vitamin D supplementation in patients with diagnosed MRONJ did not significantly prevent complications, such as wound dehiscence, incomplete wound closure, or purulent discharge (*p* = 0.079) [50].

In other clinical research, it has also been shown that vitamin D levels influence the development of MRONJ, and an adequate dosage may protect against osteonecrosis [51,52].

A 2022 in vivo study reported that administering vitamin D in any form to mice can prevent the development of MRONJ in patients undergoing tooth extractions and zoledronate treatment, an effect also observed with antibiotics or anti-inflammatory agents. Their administration inhibited the increase in inflammatory cytokine levels. Moreover, vitamin D administration also inhibited osteocyte apoptosis resulting from tooth extraction and bisphosphonate therapy [53].

In the current research, no significant difference in preoperative β-CTx levels was observed between patients who developed MRONJ and those who experienced normal postoperative healing. Additionally, stratification using clinically applied β-CTx thresholds did not reveal a significant association between this biomarker and MRONJ. In the multivariate logistic regression analysis, β-CTx did not emerge as an independent predictor of MRONJ. Our results suggest that β-CTx had no predictive value in this data set and under the measurement conditions used, without excluding the possibility of potential benefits in other clinical settings or in prospective studies. This lack of statistical significance should be interpreted in the context of the high biological variability of the biomarker, the large standard deviation observed in the cohort, and the retrospective nature of the study. The literature remains controversial regarding the predictive value of β-CTx in MRONJ, with inconsistent results across studies.

In 2007, Marx et al. [23] reported that CTx is an effective tool for assessing MRONJ risk and proposed three β-CTx risk thresholds. However, subsequent studies did not support a protective threshold of >150 mg/mL for β-CTx.

The group led by Salgueiro et al. demonstrated that there was no statistical difference in the risk of MRONJ between preoperative β-CTx levels (>150 pg/mL vs. <150 pg/mL), nor did gender, duration of bisphosphonate therapy, or comorbidities influence the development of osteonecrosis. It was reported that β-CTx levels cannot be considered a preventive or predictive measure for MRONJ [28].

Also, a meta-analysis found that β-CTx was not a reliable biomarker for predicting MRONJ in oncology patients treated with bisphosphonates who underwent oral surgery. The results confirmed that the established clinical cutoff of 150 pg/mL is not useful for assessing MRONJ risk. The mean and median were 133.4 pg/mL and 122.4 pg/mL, respectively, suggesting that a cutoff of 150 pg/mL would make it difficult to differentiate patients [29]. Similar data have been reported in other meta-analyses [26,54,55,56].

Further, the data obtained on the significant association between mandibular extractions and MRONJ are consistent with the literature. The study by Heim et al. [42] reported mandibular extractions as the most common procedure among patients treated with antiresorptive medication who developed MRONJ. In 2018, Hallmer et al. identified that mandibular localization was present in 75% of MRONJ cases [57]. Therefore, the extraction site in the lower jaw is a relevant risk factor, as demonstrated by numerous studies [36,58,59].

Our results showed that MRONJ occurred in 42.86% of patients undergoing mandibular extractions, compared with 11.54% in those undergoing maxillary extractions. Some studies have shown much more considerable differences, with MRONJ developing in the lower jaw in over 71% of patients, compared to less than 22.5% in the upper jaw [60,61]. An explanation for the higher incidence in the mandibular area may be the thinner mucosa at that level and reduced vascularity [62]. These particularities underscore the need for a stricter preventive approach to procedures performed at the mandibular level.

In our study, demographic variables, including age and gender, were not significantly associated with MRONJ. This finding indicates that, although advancing age is commonly considered a general risk factor for postoperative complications, its influence on MRONJ development appears to be indirect and primarily mediated by antiresorptive therapy and metabolic factors. Similarly, the lack of significant differences between genders is consistent with recent literature indicating that gender is not an independent determinant of MRONJ risk.

The study conducted by Ekici [63] reported that gender and age are variable risk factors in the development of MRONJ. The higher prevalence was among women, which is due to the underlying disease, such as breast cancer or osteoporosis, for which antiresorptive therapy is administered, as also stated by the group of Ruggiero et al. [1]. The average age reported was 60 years, which is consistent with our study.

Ishimaru et al. [64] also reported that the average age at which MRONJ develops is 65 years and that the pathology is observed mainly in men. A meta-analysis conducted by Rodriguez-Archilla found that female gender and advanced age may be risk factors [65]. However, these variables were not determining factors in the occurrence of MRONJ.

The type of malignancy was not significantly associated with postoperative MRONJ. This may be attributed to the greater influence of antiresorptive agent exposure and the patient’s bone status than to cancer type.

In the research conducted by Ciobanu et al. [58], it was shown that over 73% of the analyzed population was diagnosed with breast or prostate carcinoma. These findings are similar to the present study, in which 33.33% of patients were diagnosed with prostate cancer and 27.78% with breast carcinoma. Another retrospective study showed a high incidence of MRONJ in patients who received antiresorptives (IV) for bone metastases associated with prostate or breast cancer [66].

In addition, the number of teeth extracted did not significantly affect the occurrence of MRONJ, suggesting that disease mechanisms may depend on local and systemic biological factors rather than the extent of the surgical intervention. However, performing multiple extractions can be invasive, decreasing the patient’s quality of life and causing complications [67].

Year after year, medical professionals are increasingly aware of the progression of MRONJ and the need for a comprehensive analysis of the factors that lead to its development.

An aspect that must be emphasized is the multifactorial nature of MRONJ. The analyzed cohort included exclusively oncological patients treated with intravenous zoledronic acid, which ensured a relative homogeneity of the type of antiresorptive exposure. According to the local protocol of the Oral and Maxillofacial Clinic in Timisoara, antiresorptive therapy was interrupted at least two months before dental extractions. This approach was adopted based on research in the specialized literature, which showed better local effects during the drug holiday, due to a decrease in osteoclastic suppression [68]. The standardization of this therapeutic approach aimed to reduce variability in the timing of the intervention relative to treatment administration. However, the actual benefit of the drug pause remains controversial. Although a complete characterization of cumulative exposure was not possible, the analyzed group showed a high degree of therapeutic uniformity.

The development of MRONJ can be influenced by a range of systemic and local factors, including cardiovascular disease, diabetes mellitus, renal dysfunction, concomitant therapies (e.g., chemotherapy, immunotherapy, hormone therapy, anticoagulant and corticosteroid treatment), and smoking, which are recognized as risk modifiers in clinical guidelines. These determinants can affect bone-healing and predispose to postoperative complications [2,69,70,71,72]. However, regarding associated diseases, opinions in the literature are controversial regarding their potential risk in MRONJ, especially regarding diabetes mellitus [73,74,75].

Although all this is known, the regression model did not incorporate several clinical factors recognized as risk modifiers, given the relatively small number of cases (18 cases of MRONJ). Including more variables would have increased the risk of model overloading and instability in statistical estimates.

In addition, in recent years, the relationship between dental implants and the incidence of MRONJ in patients receiving antiresorptive drugs has been studied. Available data indicate that dental implants do not pose a risk of developing MRONJ in patients taking low doses of antiresorptive medication. Dental implants are not directly associated with MRONJ, whereas tooth extractions have been shown to increase the risk of MRONJ in patients receiving antiresorptive drugs [76]. There is also limited evidence that antiresorptive medication reduces dental implant failure. However, bisphosphonates may increase the risk of MRONJ in patients with osteoporosis and dental implants [77].

The current research findings have clinical implications for the management of oncologic patients undergoing dental extractions while receiving antiresorptive therapy. Preoperative screening for vitamin D deficiency is a simple, cost-effective way to detect patients at high risk of MRONJ. Correcting vitamin D deficiency before invasive dental procedures may improve bone healing. In addition, identifying the mandible as a high-risk site for MRONJ underscores the importance of careful postoperative monitoring. Integrating both metabolic and anatomical factors into preoperative assessment may decrease the risk of complications and enhance clinical outcomes.

This study has several strengths. It focuses on a well-defined, high-risk oncology group relevant to everyday clinical practice. The researchers included both metabolic biomarkers and clinical procedure factors in a comprehensive predictive model. The finding that vitamin D deficiency is an independent predictor provides directly applicable clinical value, and the use of ROC analysis offers an objective assessment of the model’s performance and clinical applicability.

However, it has some limitations that should be considered. The relatively small sample size and retrospective design did not allow for a complete evaluation of all factors possibly associated with the development of MRONJ. Incomplete characterization of antiresorptive exposure and absence of clinical variables (comorbidities, concomitant therapies) may represent potential sources of residual confounding. An additional limitation of the study concerns the length of the follow-up period. Although the 8-week interval is consistent with the case definition of MRONJ given by AAOMS, a longer follow-up period (e.g., 12–24 weeks) might allow for a more complete assessment of lesion progression and reduce the risk of potential misclassification. Future prospective studies with larger participant cohorts and additional clinical variables are needed to validate and extend these findings.

## 5. Conclusions

The results demonstrated that a preoperative vitamin D level < 30 µg/L is an independent and good predictor of MRONJ after dental extractions in oncological patients receiving antiresorptive medication. Meanwhile, β-CTx levels were not significantly associated in this dataset. Furthermore, mandibular extraction was identified as a significant risk factor for MRONJ. The multivariable predictive model clearly separated the different outcomes. This shows that combining metabolic and anatomical factors into preoperative risk assessment can be effective in clinical settings. The results highlight the importance of optimizing vitamin D levels before invasive dental procedures in high-risk patients, while recognizing that further prospective studies are needed to confirm these findings.

## Figures and Tables

**Table 1:** Baseline characteristics of the study population (N = 61).

Variables	Population
Age (years), mean ± SD (range)	64.84 ± 9.41 (42–82)
Sex, N (%)	
Women	37 (60.7)
Men	24 (39.3)
Malignancies, N (%)	
Brain cancer	5 (8.2)
Breast cancer	25 (40.98)
Broncho-pulmonary cancer	2 (3.28)
Kidney cancer	5 (8.2)
Multiple myeloma	2 (3.28)
Prostate cancer	19 (31.15)
Stomach cancer	3 (4.92)
Location, N (%)	
Mandible	35 (57.38)
Maxilla	26 (42.62)
Teeth extracted, mean ± SD	2.69 ± 1.65
Metabolic profile	
β-CTx (pg/mL), median (Q1–Q3)	332 (183–511)
25-hydroxyvitamin D (µg/L), median (Q1–Q3)	27.07 (20.62–35.17)
8 weeks follow-up	
Normal healing, N (%)	43 (70.49)
Non–healing (MRONJ at 8 weeks), N (%)	18 (29.51)

SD: standard deviation; Q1–Q3: first and third quartiles.

**Table 2:** Age distribution according to postoperative outcome at 8-week follow-up.

	8 Weeks Follow-Up		
Age	MRONJ (n = 18)	Normal Healing (n = 43)	*p*-Value
Mean ± SD	63.89 ± 10.16	65.23 ± 9.17	
Median (Q1–Q3)	64 (55–74)	68 (59–73)	0.645

SD: standard deviation; Q1–Q3: first and third quartiles. The Mann–Whitney U test was used.

**Table 3:** Number of extracted teeth according to postoperative outcome at 8-week follow-up.

	8 Weeks Follow-Up		
Teeth Number	MRONJ (n = 18)	Normal Healing (n = 43)	*p*-Value
Mean ± SD	2.667 ± 1.534	2.698 ± 1.712	
Median (Q1–Q3)	2 (1.25–4)	2 (1.5–3)	0.929

SD: standard deviation; Q1–Q3: first and third quartiles. The Mann–Whitney U test was applied.

**Table 4:** Preoperative serum β-CTx (pg/mL) and 25-OH-vitamin D (μg/L) levels according to clinical outcome at 8-week follow-up.

	Group	Mean ± SD	Median (Q1–Q3)	*p*-Value
β-CTx	MRONJ	370.61 ± 184.38	367 (287.8–499)	0.621
	Normal healing	353.16 ± 226.35	321 (170–494)	
25-OH-vitamin D	MRONJ	22.16 ± 6.85	20.48 (17.52–25.65)	0.001 *
	Normal healing	31.35 ± 10.59	31.76 (22.01–37.44)	

SD: standard deviation; Q1–Q3: first and third quartiles. The Mann–Whitney U test was used. * *p* < 0.05 was considered statistically significant.

**Table 5:** Association between preoperative 25-OH-vitamin D and β-CTx levels with MRONJ occurrence at 8-week follow-up.

		8 Weeks Follow-Up			
Variable	Category	MRONJ, n (%)	Normal Healing, n (%)	OR (95% CI)	*p*-Value
25-OH-vitamin D	>30 μg/L	3 (11.11)	24 (88.89)	Reference	-
	<30 μg/L	15 (44.12)	19 (55.88)	6.32 (1.59–25.05)	0.006 *
β-CTx	>150 pg/L	15 (31.25)	33 (68.75)	Reference	-
	<150 pg/L	3 (23.08)	10 (76.92)	0.66 (0.16–2.75)	0.737

OR: odds ratio; CI: confidence interval. Fisher’s exact test was applied. * *p* < 0.05 was considered statistically significant.

**Table 6:** Multivariate logistic regression model of preoperative metabolic biomarkers associated with MRONJ occurrence.

Variable	Category	OR	95% CI	*p*-Value
25-OH-vitamin D	<30 µg/L vs. >30 µg/L	6.33	1.59–25.19	0.009
β-CTx	<150 pg/mL vs. >150 pg/mL	0.65	0.14–2.97	0.579

OR: odds ratio; CI: confidence interval. Multivariate logistic regression analysis was performed. *p*-values were calculated using the Wald test.

**Table 7:** Comprehensive multivariate logistic regression model for predictors of MRONJ occurrence.

Variable	Category	OR	95% CI	*p*-Value
25-OH-vitamin D	<30 µg/L vs. >30 µg/L	8.74	1.96–39.03	0.005
Extraction site	mandible vs. maxilla	7.94	1.75–35.99	0.007
β-CTx	<150 pg/mL vs. >150 pg/mL	0.82	0.15–4.47	0.815
Age	per year increase	0.99	0.93–1.06	0.866

OR: odds ratio; CI: confidence interval. Multivariate logistic regression analysis was performed. *p*-values were obtained using the Wald test.

## Data Availability

The data presented in this study are available on request from the corresponding author. The data are not publicly available due to restrictions related to the privacy of the patients and the funding protocol.

## Abbreviations

The following abbreviations are used in this manuscript:

MRONJ	Medication-related osteonecrosis of the jaw
AAOMS	American Association of Oral and Maxillofacial Surgeons
β-CTx	Serum β-C-terminal telopeptide cross-link
25-OH-vitamin D	25-hydroxyvitamin D
SD	Standard deviation
Q1–Q3	First and third quartiles
OR	Odds ratio
CI	Confidence interval
ROC	Receiver operating characteristic
AUC	Area under the curve
